# Khat Chewing and Lipid Profile in Human and Experimental Animals

**DOI:** 10.1155/2021/6001885

**Published:** 2021-12-24

**Authors:** Mohammed A. Al-Duais, Yahya S. Al-Awthan

**Affiliations:** ^1^Department of Biochemistry, Faculty of Science, University of Tabuk, Tabuk, Saudi Arabia; ^2^Biochemistry Unit, Chemistry Department, Faculty of Science, Ibb University, Ibb, Yemen; ^3^Department of Biology, Faculty of Science, University of Tabuk, Tabuk, Saudi Arabia; ^4^Department of Biology, Faculty of Science, Ibb University, Ibb, Yemen

## Abstract

**Background:**

*Catha edulis*, also known as khat or qat, is a plant that grows in East Africa and southern Arabia. Several millions of people chew the leaves and twigs of khat plant for their central stimulating amphetamine-like effects. Khat chewing is becoming more common in Europe and the United States, owing to worldwide migration.

**Objective:**

This review examines the khat ingredients, global prevalence, and legal status as well as its effects on lipid profile. *Methodology*. A literature search was performed using PubMed, Scopus, and Google Scholar to collect information within articles published up to April 2021 with the goal of identifying relevant studies. The proposed mechanisms of cathinone effects on total cholesterol and triglyceride were also discussed.

**Conclusion:**

The khat chewing habit is considered as a serious economic and health issue that needs specialized programs to assist those people to quit or reducing this habit.

## 1. Introduction

**Figure 1:**
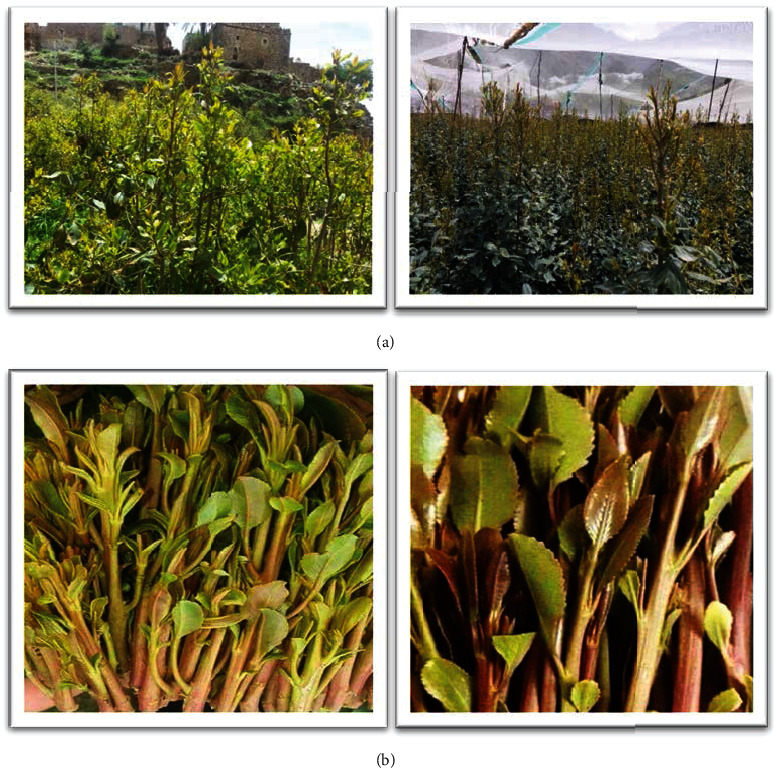
Khat images. (a) Khat plant; (b) fresh leaves and twigs for chewing purpose.

Khat (*Catha edulis* Forsk) is a perennial plant commonly grown in Yemen, Ethiopia, and other horn African countries [1]. Chewing khat is a social and cultural habit practiced by Yemeni and East African peoples [2]. The tender, reddish-green leaves and the young shoots (Figure 1) are chewed for several hours daily to get the desired effects [3]. Generally, people chew khat for its stimulant effects on nervous system and reducing fatigue and restore mental and physical activity [4]. It has been reported that Ethiopia was the original country of khat cultivation and then was spread to Somalia, Djibouti, Kenya, and Yemen [5].

Khat is known by many local names: mirra in Kenya, qat and khat in Yemen, qaadorjaad in Somalia, and chat in Ethiopia [6]. It was reported that khat chewing prevalence in Yemeni general population is 67% [7]. A recent study conducted in Saudi Arabia described the high prevalence of khat chewing in Jazan region especially by diabetic patients [8]. The estimated global khat consumers are about 20 million [9]. Even though there are a huge number of studies reporting the psychological and physiological effects of khat on different body systems, available data exploring khat effects on lipid profile are scarce. Taking into consideration that several millions of people globally chew khat on daily basis, it is likely that khat adverse health effects including effects on lipid profile will be seen throughout the globe. Therefore, it is important for physicians with different specialists to know the khat effects on lipid profile. Therefore, this review deals with the habit of khat use, its biodiversity, phytochemistry, cultivation, harvesting, prevalence, and legal status. The khat effects on lipid profile including total cholesterol (TC), high-density lipoprotein cholesterol (HDL-C), low-density lipoprotein cholesterol (LDL-C), and triglycerides (TG) will be also discussed. Other negative health impacts connected with khat chewing such as oral health effects, nervous effects, and obesity are discussed elsewhere [9].

**Figure 2:**
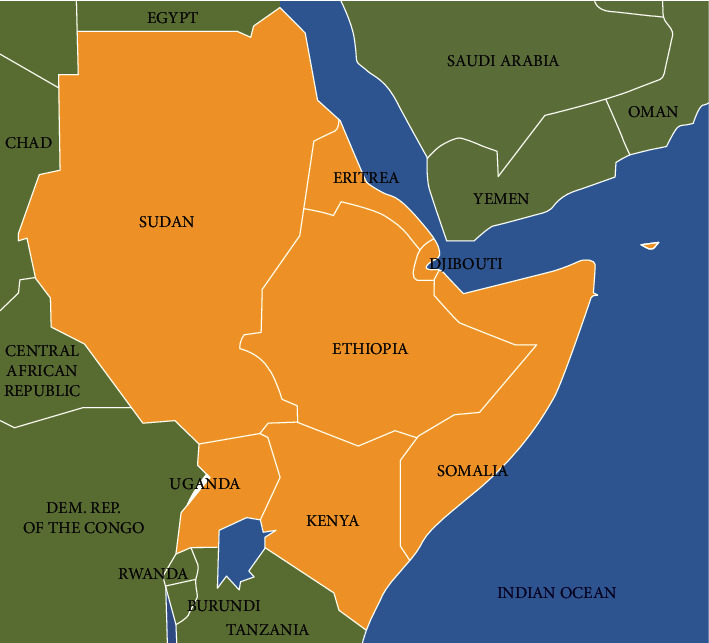
The map of Horn Africa and Yemen. Source: https://commons.wikimedia.org/wiki/File:Horn_of_Africa_map.png.

The khat plant varies in phenotype and genotype according to the geographical area and environment where each type grows. In Yemen, more than 40 genotypes were specified [10]. Generally, the type and local name of khat is derived from the name of the region in which it is grown and/or sold. In Yemen for instance, the most famous species are Al-Shami cultivated in Hajah and Al-Mahwit governates, Al-Sawtty in Sana, Amran, and Dhamar provinces, Al-Sabri in Taiz, and Al-Yafei in Yafeh regions; in the same manner, Harari type in Ethiopia, Magdeshu type in Somalia, and Gachoka type in Kenya (Figure 2) [4]. The fresh leaves of each type differ in shape, color, and taste. The lamina of leaves is fleshy with many shapes. Furthermore, the content of alkaloids and other compounds is also different [11].

### 1.1. Phytochemistry of Khat

**Figure 3:**
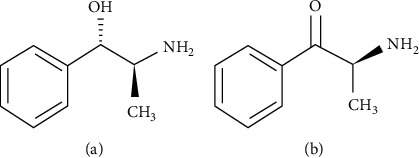
The structures of (a) cathine and (b) cathinone.

Khat leaves contain many various compounds: alkaloids, flavonoids, tannins, glycosides, vitamins, terpenoids, minerals, and more than ten amino acids [12]. Both cathinone and cathine are the most important psychoactive amphetamine-like alkaloids in khat, with the predominant of cathinone in fresh leaves and twigs [13]. The chemical structures of the two alkaloids are shown in Figure 3. By drying, storing, and maturing cathinone is converted into cathine [14]. On the contrary, tannins and phenolic compounds such as flavanols and kaempferol were obtained in significant quantities [15]. Despite the presence of different compounds in khat leaves and twigs, the effects of khat chewing are clearly caused by cathinone, cathine, and tannins [16].

### 1.2. Cultivation, Harvesting, and Consumption

In Yemen, khat is grown in all regions of the central and western highlands, in which most of the Yemeni population is concentrated. In this region, the most common famous types of Yemeni khat are cultivated [17]. The coastal and desert areas are not suitable climatically for cultivation of khat (Figure 2). In Ethiopia, khat is cultivated in almost highlands in the middle altitudes between 1500 and 2100 meters, and the land used to khat cultivate is greatly increased [18]. In Kenya, the cultivation of khat is also widespread especially in North Eastern Province [19]. Regarding khat gathering, usually in the early morning time, the tender, reddish-green leaves are cut together with the twigs by khat pickers who are usually khat growers and sellers and collects them in small or large bundles. Then, these bundles are transferred to the market to be sold to consumers on the same day, and if the sale is delayed until the next day, this reduces the quality and price of the khat due to the conversion of psychoactive cathinone to less active cathine, and this explains the preference of khat users for fresh leaves and twigs [20, 21]. Khat session started usually between 1 and 2 PM daily when the men gather in one place called Majlis or Diwan and start to pick fresh leaves and twigs and chew them on one side to allow juice to be absorbed via the oral mucosa, or through the gastrointestinal tract after swallowing it with well-chewed leaves, adding periodically fresh leaves and twigs to obtain euphoria [8, 22]. Normally, each khat consumer chewed between 200 and 500 grams of khat daily.

## 2. Prevalence and Legal Status of Khat

Yemen, Ethiopia, Somalia, Djibouti, Tanzania, and Kenya are among the countries where khat chewing is legal and prevalent [9, 23]. In these countries, there are no restrictions on the consumption of khat neither in terms of age nor gender. In addition, there is an increase in khat consumption among children and women [24, 25]. Recent reports showing the spread of khat use among immigrants from the aforementioned countries to other countries Europe, Australia, Canada, and the USA [14, 26, 27]. Moreover, khat use has been observed among host western teenagers [14]. Khat is illegal in Canada, the USA, the UK, Germany, France, and other European Countries [9].

## 3. Methodology

A literature search was performed using PubMed, Scopus, and Google Scholar to collect information within articles published from 2000 until April 2021 with the goal of identifying relevant studies and describing and consolidating observational and intervention data that provided khat chewing and lipid profile such as TC, LDL-C, HDL-C, and triglycerides in human and animals. Only articles written in English language were included in the search. The following combinations of the keywords were used: khat, qat, TC, TG, HDL-C, and LDL-C.

## 4. Discussion

Khat consumption is one of the most important causes that lead to a large number of health disorders for individuals. There are several studies dealing with the effects of khat on different parts of the human body; however, there are no previous revision on the effect of khat consumption on lipid profile.

The effects of khat on lipid profile are still inconclusive, and this phenomenon can be explained by the following factors. First, the khat used in those studies was harvested and collected from different geographical areas, and this could affect the concentrations of cathine, cathinone, and other khat components. Secondly, the khat effects were almost attributed to the cathine and cathinone but the effects of other khat components such as flavonoids, terpenoids, and minerals were neglected. It is very important to perform more studies reporting in detail the role of those components. Furthermore, pesticides widely used in khat agriculture can cause toxic effects, which needs more studies. Third, the results of the selected published articles were obtained according to a restricted number of prospective and case-control studies. As a result, comprehensive studies are urgently needed to investigate the role of khat chewing in the metabolism of cholesterol, triglycerides, and lipoproteins. Finally, khat chewing habit is considered a serious economic and health issue that needs specialized programs to assist those people to quit or reduce this habit.

### 4.1. Lipid Profile and Cardiovascular Disorders

Lipid profile refers to the levels of total cholesterol (TC), low-density lipoprotein cholesterol (LDL-C), high-density lipoprotein cholesterol (HDL-C), and triglycerides (TG) [28]. Dyslipidemia is a term referring to the disorders of lipoprotein metabolism and is clinically characterized by elevated plasma levels of TC, LDL-C, and TG as well as decreased levels of HDL-C [29]. Dyslipidemia increases the risk of atherosclerosis and cardiovascular disease (CVD) [30, 31]. It has been reported that CVD is one of the leading causes of mortality and morbidity worldwide [32]. For instance, CVD was the 5^th^ cause of mortality in the USA in 2016 [33] and causes 49% of mortality in Europe [34]. The serum lipid profile is influenced by many dietary and habitual factors [35].

### 4.2. The Effects of Khat on Total Cholesterol (TC) and Low-Density Lipoprotein Cholesterol (LDL-C)

Studies reporting the effects of khat chewing on TC and LDL-C levels are conflicting. The study of Al-Zubiri et al. (*n* = 107) demonstrated nonsignificant lowered effects of khat on fasting TC and LDL-C levels in khat chewers [36]. They also found the same effects after 2 hrs of khat chewing session in khat chewers comparing with nonkhat chewers [36]. Additionally, the study of Al-Akwa (*n* = 60) reported similar reducing effects among healthy male khat users [37]. Moreover, a study carried out in Jazan region in the Kingdom of Saudi Arabia (*n* = 100) confirmed the decreased effects of khat chewing on TC and LDL-C among Saudi khat consumers [38]. In one recent Ethiopian cross-sectional-based study (*n* = 9800), it has been found a significantly low median of TC and LDL-C in khat consumers compared with nonkhat consumers [39]. The study of Masoud et al. which included only women, demonstrated similar reducing effects of khat on TC and LDL-C in female khat users comparing with nonkhat users [40]. The results of a study conducted in 2017 on diabetic patients (*n* = 260) was in an agreement with the reducing effects of khat. Their results showed lowered levels of TC and LDL-C in khat chewing diabetics than nonkhat chewing (181.3 ± 33.2 mg/dl and 109 ± 7 mg/dl versus 196.3 ± 44.7 mg/dl and 117.4 mg/dl, respectively) [41]. Those authors attributed the reducing cholesterol level to an increase in cyclic adenosine monophosphate (c-AMP), which inhibits cholesterol synthesis. Furthermore, the raised levels of adrenocorticotrophic hormone (ACTH) in khat chewers enhance the c-AMP production [39, 42]. On the contrary, the reduced effects of khat on TC and LDL-C were inconsistent with the findings of other human studies. For instance, Al-Motarreb et al. (*n* = 157) reported higher levels of TC by twice the time among khat chewing patients having acute myocardial infarction than nonkhat chewer patients [43]. A large cohort study including 8176 acute coronary syndrome (ACS) patients from six Arabic neighboring countries located in the Arabian Peninsula (Kuwait, Qatar, Bahrain, United Arab Emirates, Oman, and Yemen) estimated high levels of TC in ACS khat chewers (*n* = 934) than ASC nonkhat chewers (*n* = 7242) (224.4 ± 116.1 mg/dl vs. 1 ± 77.4 mg/dl, respectively, *P* < 0.001) [44]. A more recent study on healthy university Yemeni male students (*n* = 360) noted the same observation of increased serum levels of TC and LDL-C in khat chewers (*n* = 283) than nonkhat chewers (*n* = 77) [45]. Another human study in Yemen (*n* = 70) offered a nonsignificant increase of TC among khat users comparing with nonkhat users [46]. Also, another human study (*n* = 100) found that the plasma levels of TC in males were not affected by chewing of 200 g and 400 g of fresh khat leaves after 4 hours of khat session [47]. Moreover, the presence of higher cholesterol levels in the saliva of khat consumers by 3.9 times greater than nonkhat consumers has been observed [42]. Taking into account that the saliva cholesterol level is correlated with the serum cholesterol level [48]. Saliva cholesterol may be transported from serum to saliva by ultrafiltration process via gap junctions located between cells of secretory units [42].

### 4.3. The Effects of Khat on High-Density Lipoprotein Cholesterol (HDL-C)

HDL-C is referred to as “good cholesterol” because of its role in the transport of excess cholesterol from peripheral tissues to the liver, preventing atherosclerotic plaque formation [49]. The increase of HDL-C level reduces the risk of CVD [50]. It has been estimated by many authors that khat reduces HDL-C levels. For example, a study established in 2009 reported significant decreased in HDL-C level in khat chewers compared to nonkhat chewers (24.72 ± 4.48 mg/dl versus 33.34 ± 1.83 mg/dl) [37]. A similar finding was obtained by Alam et al. who demonstrated the presence of hypoalphalipoproteinemia among Saudi khat users [38]. According to a recent study, HDL-C was low in 83% of khat chewers versus 75.3% in nonkhat chewers [45]. They also found a predominant of isolated low HDL-C in khat consumers than nonkhat consumers (25.1% versus 15.6%, respectively). Similar data of lowered HDL-C in khat users was estimated by one Ethiopian study [39]. On the contrary, a study conducted in Yemen demonstrated higher fasting plasma levels of HDL-C by 15% in khat consumers than nonkhat consumers [36].

### 4.4. The Effects of Khat on Triglycerides

Studies reporting the effects of khat on serum TG are different and inconsistent. In one Yemeni study in 2003, it has been shown increasing levels of fasting TG in khat chewers than nonkhat chewers (92.77 ± 49.26 versus 85.15 ± 36.73 mg/dl) [36]. The striking point in this study was the decreased levels of TG in consumers after 2 hrs. of khat chewing session [36]. In patients with acute myocardial infarction, it was noticed higher TG levels in khat chewer patients than in nonkhat chewers [43]. Additionally, Ali et al. demonstrated higher serum TG levels in ACS khat user patients than nonkhat user patients (288.7 ± 525.0 mg/dl vs. 210.0 ± 787.5 mg/dl; *P* < 0.003) [44]. In the same manner, the study conducted by Al-Akwa established a significant increase of TG levels in nonkhat users concerning nonkhat users [37]. Furthermore, one Saudi study reported a two times increase in TG levels among khat chewers than nonkhat chewers [38]. On the contrary, these findings were incompatible with other studies [47]. They showed significant diminished TG levels by 30% and 36.7% after chewing 200 g and 400 g of khat leaves, respectively, in khat users in comparison with nonkhat users after 4 hours of starting the khat session. However, in this study, the one measurement of TG may not reflect the real effect of khat chewing. A recent study demonstrated a high significant decrease in TG levels in khat consumers comparing with nonkhat consumers [45]. The nonsignificant reducing effect of khat was also found among diabetic khat consumers [41]. Regarding the reducing khat effects on TG, it could be due to the effect of amphetamine-like cathinone, which enhances lipolysis via *β*-adrenergic receptors [51]. This finding was in close similarity with Zhang et al. who reported the reducing levels of TC and TG among methamphetamine dependence patients was published [52]. The hypotriglyceridemic effect of khat appears to be due to the synergistic effects of its various constituents rather than cathinone only. It has been estimated the inhibition of lipid intestinal absorption by tannins [53]. On the other hand, one Ethiopian study has concluded the absence of differences in TG levels in khat chewers in comparison with nonchewers [39]. The disparities in those findings could be related to the different types and quantities of khat consumed and the duration of khat sessions. The age, gender, and individual health state of each khat consumer also may be related to the inconsistency of the results.

### 4.5. Animals and In Vitro Studies

It has been demonstrated the reducing effects of khat on TC and TG in rabbits throughout 6 months of animal treatment using different doses [54]. Those authors also found a significant increase of HDL-C after 6 months of feeding khat containing diet [54]. The inverse relationship between TG and HDL-C has been observed [55]. A study conducted later on Wistar Ottawa Karlsburg RT1^u^ rats estimated the lowering effects of khat on TG levels [56]. The authors concluded that khat intake had no significant effect on serum TC, VLDL-C, LDL-C, and HDL-C in rats [56]. Another study showed significant reducing effects of khat on TC, LDL-C, and HDL-C levels and elevated effect on TG levels in rabbits [57]. A more recent study on mice reported the decreased levels of TC and LDL-C in the group of mice treated with pure cathinone only rather than khat treated groups [58]. Those authors also concluded the unaffected levels of TC, LDL-C, and HDL-C after treatment with khat extracts for 8 weeks. In addition, the hypotriglyceridemic effect of khat on mice was also observed [58]. One preliminary *in vitro* study estimated the reducing effects of khat extracts on TC and TG [59]. They proposed the enhancement of lipolysis by khat extract.

## 5. Limitations of the Study

This review was conducted based on a restricted number of human and animal studies. Therefore, comprehensive future studies are necessary to investigate the role of khat components in the metabolism of cholesterol, triglycerides, and lipoproteins.

## 6. Conclusion

Khat is a widely distributed plant in southern Arabia and the Horn of Africa. The chewing of khat is a common habit among people of those communities. According to the previous studies, it was found that the effect of khat consumption on lipid profile indicators is somewhat conflicting, as some studies indicated that khat raises the levels of total cholesterol and LDL-C while other studies have indicated opposite results. In addition, the majority of reported articles found a reduced effect of khat chewing on HDL-C while only one study indicated an increased effect. Also, the results of previous reports showed that there is a conflict in the effect of khat chewing on triglyceride levels, as most studies showed an increase in the level of triglycerides, while other studies indicated low levels. We conclude that the effects of khat consumption on lipid profile are still inconclusive and need more investigation.

## References

[1] Girma T., Mossie A., Getu Y. (2015). Association between body composition and khat chewing in Ethiopian adults. *BMC Research Notes*.

[2] Alshagga M. A., Alshawsh M. A., Seyedan A. (2017). Khat (Catha edulis) and obesity: a scoping review of animal and human studies. *Annals of Nutrition & Metabolism*.

[3] Orlien S. M. S., Sandven I., Berhe N. B. (2018). Khat chewing increases the risk for developing chronic liver disease: a hospital-based case–control study. *Hepatology*.

[4] Alsalahi A., Alshawsh M. A., Mohamed R. (2016). Conflicting reports on the role of the glycemic effect of _Catha edulis_ (Khat): A systematic review and meta-analysis. *Journal of Ethnopharmacology*.

[5] Gebrie A., Alebel A., Zegeye A., Tesfaye B. (2018). Prevalence and predictors of khat chewing among Ethiopian university students: a systematic review and meta-analysis. *PLoS One*.

[6] Abdeta T., Tolessa D., Adorjan K., Abera M. (2017). Prevalence, withdrawal symptoms and associated factors of khat chewing among students at Jimma University in Ethiopia. *BMC Psychiatry*.

[7] Al-Abed A. A. A., Sutan R., Al-Dubai S. A. R., Aljunid S. M. (2014). Family context and khat chewing among adult Yemeni women: a cross-sectional study. *BioMed Research International*.

[8] Badedi M., Darraj H., Hummadi A. (2020). Khat chewing and type 2 diabetes mellitus. *Diabetes, Metabolic Syndrome and Obesity: Targets and Therapy*.

[9] Patel N. B. (2019). Khat (_Catha edulis_Forsk) - And now there are three. *Brain Research Bulletin*.

[10] Al-Motarreb A., Baker K., Broadley K. J. (2002). Khat: pharmacological and medical aspects and its social use in Yemen. *Phytotherapy Research*.

[11] Gambaro V., Arnoldi S., Colombo M. L., Dell’Acqua L., Guerrini K., Roda G. (2012). Determination of the active principles of _Catha Edulis_ : Quali-quantitative analysis of cathinone, cathine, and phenylpropanolamine. *Forensic Science International*.

[12] Engidawork E. (2017). Pharmacological and toxicological effects of Catha edulis F. (khat). *Phytotherapy Research*.

[13] Tembrock L. R., Broeckling C. D., Heuberger A. L., Simmons M. P., Stermitz F. R., Uvarov J. M. (2017). Employing two-stage derivatisation and GC–MS to assay for cathine and related stimulant alkaloids across the Celastraceae. *Phytochemical Analysis*.

[14] Pendl E., Pauritsch U., Kollroser M., Schmid M. G. (2021). Determination of cathinone and cathine in Khat plant material by LC-MS/MS: Fresh vs. dried leaves. *Forensic Science International*.

[15] Al-Maweri S. A., Warnakulasuriya S., Samran A. (2018). Khat (Catha edulis) and its oral health effects: an updated review. *Journal of Investigative and Clinical Dentistry*.

[16] Samue Kindie M. A. (2015). Adverse health effects of Khat: a review. *Family Medicine & Medical Science Research*.

[17] Zahran M. A., Khedr A., Dahmash A., El-Ameir Y. A. (2014). Qat farms in Yemen: ecology, dangerous impacts and future promise. *Egyptian Journal of Basic and Applied Sciences*.

[18] Cochrane L., O’Regan D. (2016). Legal harvest and illegal trade: trends, challenges, and options in khat production in Ethiopia. *The International Journal on Drug Policy*.

[19] Aden A., Dimba E. A. O., Ndolo U. M., Chindia M. L. (2006). Socio-economic effects of khat chewing in north eastern Kenya. *East African Medical Journal*.

[20] Yi R., Zhao S., Lam G., Sandhu J., Loganathan D., Morrissey B. (2013). Detection and elimination profile of cathinone in equine after norephedrine (Propalin®) administration using a validated liquid chromatography-tandem mass spectrometry method. *Analytical and Bioanalytical Chemistry*.

[21] Laminal S. (2010). Khat (Catha edulis): the herb with offcio-legal, socio-cultural and economic uncertainty. *South African Journal of Science*.

[22] Kassim S., Jawad M., Croucher R., Akl E. A. (2015). The epidemiology of tobacco use among khat users: a systematic review. *BioMed Research International*.

[23] Mahfouz M. S., Rahim B. E. E., Solan Y. M., Makeen A. M., Alsanosy R. M. (2015). Khat chewing habits in the population of the Jazan region, Saudi Arabia: prevalence and associated factors. *PloS one*.

[24] Al-Duais M. A., Al-Awthan Y. S. (2019). Prevalence of dyslipidemia among students of a Yemeni university. *Journal of Taibah University Medical Sciences*.

[25] Al-Motarreb A., Al-Habori M., Broadley K. J. (2010). Khat chewing, cardiovascular diseases and other internal medical problems: the current situation and directions for future research. *Journal of Ethnopharmacology*.

[26] Kassim S. (2020). The impact of protective psychosocial factors on khat chewing among male medical and dental future health-care providers in Yemen. *Journal of Dental Sciences*.

[27] Kwon N. J., Han E. (2019). A review of drug abuse in recently reported cases of driving under the influence of drugs (DUID) in Asia, USA, and Europe. *Forensic Science International*.

[28] Debecho D. A., Abebe Y., Seifu D., Tolessa T. (2017). Effect of crude extract of khat (Catha Edulis) on the plasma glucose level of normoglycemic and STZ induced type 2 diabetic rats. *International Journal of Health Sciences and Research*.

[29] Cho S., Han E. (2018). Association of breastfeeding duration with dyslipidemia in women aged over 20 years: Korea National Health and Nutrition Examination Survey 2010-2014. *Journal of Clinical Lipidology*.

[30] Darroudi S., Saberi-Karimian M., Tayefi M. (2018). Prevalence of combined and noncombined dyslipidemia in an Iranian population. *Journal of Clinical Laboratory Analysis*.

[31] Expert panel on integrated guidelines for cardiovascular health and risk reduction in children and adolescents (2011). Expert panel on integrated guidelines for cardiovascular health and risk reduction in children and adolescents: summary report. *Pediatrics*.

[32] Khan M. A., Hashim M. J., Mustafa H. (2020). Global epidemiology of ischemic heart disease: results from the global burden of disease study. *Cureus*.

[33] Soliman G. A. (2019). Dietary fiber, atherosclerosis, and cardiovascular disease. *Nutrients*.

[34] Francula-Zaninovic S., Nola I. A. (2018). Management of measurable variable cardiovascular disease’ risk factors. *Current Cardiology Reviews*.

[35] Lazo-Porras M., Bernabe-Ortiz A., Quispe R. (2017). Urbanization, mainly rurality, but not altitude is associated with dyslipidemia profiles. *Journal of Clinical Lipidology*.

[36] Al-Zubairi A., Al-Habori M., Al-Geiry A. (2003). Effect of _Catha edulis_ (khat) chewing on plasma lipid peroxidation. *Journal of Ethnopharmacology*.

[37] Al-Akwa A. (2009). The effect of khat on the levels of cortisol and lipid profile in healthy khat Chewres. *Bulletin of Egyptian Society for Physiological Sciences*.

[38] Alam M. S., Ali A., Jerah B. I. N., Nabi G., Husain Q. (2014). Effect of khat (Catha Edulis) consumption on the functions of liver, kidney and lipid profile in male population of Jazan Region of Kingdom of Saudi Arabia. *Ijans*.

[39] Tadele A., Getachew T., Defar A., Taye G., Molla G. (2015). Effect of khat consumption on blood biochemical parameters: evidences from the Ethiopian non communicable diseases STEPS survey. *Ethiopian Journal of Public Health and Nutrition*.

[40] Masoud A. M., Al-shehari B. A., Al-hattar L. N., Altaezzi M. A., Al-khadher W. A., Zindal Y. N. (2012). Alterations in antioxidant defense system in the plasma of female khat chewers of Thamar City, Yemen. *Jordan Journal of Biological Sciences*.

[41] Atef Z., Bamashmos M., Alghazali G. (2017). Effect of Qat on the level of blood glucose and lipids among Yemeni patients with type 2 diabetes. *Egypt. J. Obesity, Diabetes Endocrinol.*.

[42] Masoud A., Al-Qaisy A., Al-Faqeeh A. (2016). Decreased antioxidants in the saliva of khat chewers. *The Saudi Journal for Dental Research*.

[43] Al-Motarreb A., Al-Kebsi M., Al-Adhi B., Broadley K. J. (2002). *Khat chewing and acute myocardial infarction*.

[44] Ali W. M., Zubaid M., Al-Motarreb A. (2010). Association of khat chewing with increased risk of stroke and death in patients presenting with acute coronary syndrome. *Mayo Clinic Proceedings*.

[45] Al-Duais M. A., Al-Awthan Y. S. (2019). Association between qat chewing and dyslipidaemia among young males. *Journal of Taibah University Medical Sciences*.

[46] Al Ashwal R. H., Al-Maqtari M. A., Naji K. M., Al-wsabai N. A., Alhazmi S. M. (2013). Potential health effects of daily khat leaves chewing: study on the biochemical blood constituents changes among adults in Sana’a City, Yemen. *International Journal of Biochemistry and Biotechnology*.

[47] Al-Dubai W., AL-Habori M., Al-Geiry A. (2006). Human khat (_Catha edulis_) chewers have elevated plasma leptin and nonesterified fatty acids. *Nutrition Research*.

[48] Singh S., Ramesh V., Oza N., Balamurali P. D., Prashad K. V., Balakrishnan P. (2014). Evaluation of serum and salivary lipid profile: a correlative study. *Journal of Oral and Maxillofacial Pathology*.

[49] Ling Y., Jiang J., Wu B., Gao X. (2017). Serum triglyceride, high-density lipoprotein cholesterol, apolipoprotein B, and coronary heart disease in a Chinese population undergoing coronary angiography. *Journal of Clinical Lipidology*.

[50] Talbot C. P. J., Plat J., Ritsch A., Mensink R. P. (2018). Determinants of cholesterol efflux capacity in humans. *Progress in Lipid Research*.

[51] Valente M. J., Guedes de Pinho P., de Lourdes Bastos M., Carvalho F., Carvalho M. (2014). Khat and synthetic cathinones: a review. *Archives of Toxicology*.

[52] Zhang M., Lv D., Zhou W. (2017). The levels of triglyceride and total cholesterol in methamphetamine dependence. *Medicine (Baltimore)*.

[53] Yang C. S., Zhang J., Zhang L., Huang J., Wang Y. (2016). Mechanisms of body weight reduction and metabolic syndrome alleviation by tea. *Molecular Nutrition & Food Research*.

[54] Al-Habori M., Al-Mamary M. (2004). Long-term feeding effects of _Catha edulis_ leaves on blood constituents in animals. *Phytomedicine*.

[55] Hata Y., Nakajima K. (2000). Life-style and serum lipids and lipoproteins. *Journal of Atherosclerosis and Thrombosis*.

[56] Mahmood S., Lindequist U. (2008). A pilot study on the effect of Catha edulis Frosk., (celastraceae) on metabolic syndrome in WOKW rats. *African Journal of Traditional, Complementary and Alternative Medicines*.

[57] ALRajhi W. I., Yousef O. M. (2013). Effects of Catha edulis abuse on glucose, lipid profiles and liver histopathology in rabbit. *Journal of Life Sciences and Technologies*.

[58] Alshagga M. A., Mohamed Z., Seyedan A., Ebling F. J. P., Alshawsh M. A. (2020). Khat (_Catha edulis_) upregulates lipolytic genes in white adipose tissue of male obese mice (C57BL/6J). *Journal of Ethnopharmacology*.

[59] Aziz H. A., Tan Y. T. F., Peh K. K., Yam M. F. (2010). Direct effect of khat and garlic extracts on blood lipids contents: Preliminary _in vitro_ study. *Obesity Research & Clinical Practice*.

